# Delivery of small interfering RNA through lyophilized natural lipid nanoparticles: effects of natural lipid selection

**DOI:** 10.1080/13880209.2025.2498169

**Published:** 2025-05-02

**Authors:** Hangjie Wang, Wei Li, Junyan Chen, Rong Chen, Yuwei Qi, Linshuang Shen, Kaidi Chen, Lewei Dai, Yuxin Sheng, An Wang, Hong Wang, Chujian Chen, Xiao Cheng, Mancang Gu

**Affiliations:** a School of Pharmaceutical Science, Zhejiang Chinese Medical University, Hangzhou, P. R. China; b Academy of Chinese Medical Sciences, Zhejiang Chinese Medical University, Hangzhou, P. R. China; c Huzhou Institute for Food and Drug Control, Huzhou, P. R. China

**Keywords:** *Coix* seed lipid, *Brucea javanica* seed lipid, soybean oil, endosomal escape, lipid droplet targeting, siRNA transfection, cryodesiccation

## Abstract

**Context:**

Lipid nanoparticles (LNPs) are the primary non-viral vectors for siRNA delivery. However, synthetic lipids face issues, such as low lysosomal escape efficiency and high cost.

**Objective:**

This study aimed to use three natural lipids to construct LNPs, optimize their preparation and freeze-drying processes, and evaluate their siRNA delivery efficiency *in vitro*.

**Materials and methods:**

*Coix* seed lipid [*Coix lacryma-jobi* L. var. *mayuen* (Roman.) Stapf (Poaceae), CSL], *Brucea javanica* seed lipid [*Brucea javanica* (L.) Merr. (Simaroubaceae), BJL], and Soybean oil [*Glycine max* (L.) Merr. (Fabaceae), SO] were used to construct LNPs. The Z-average size, zeta potential, Polymer Dispersity Index, and N/P ratio of the LNPs were characterized. Transmission electron microscope was used for morphology observation and the MTS assay for cytotoxicity. Confocal laser scanning microscope assessed cell uptake, lysosomal escape, and co-localization of lipid droplets. The efficiency of siRNA knockdown was evaluated in three cells using qPCR and Western blot. The freeze-drying processes were optimized.

**Results:**

The optimal LNPs exhibited a size of 160–180 nm, zeta of 44–50 mV, and PDI of <0.2. At 200 μg/mL, the LNPs did not affect cell viability. CSL-LNPs, BJL-LNPs, and SO-LNPs reduced KRAS^G12D^ mRNA levels in AsPC-1 cells by 67.87 ± 3.89, 47.18 ± 7.65, and 42.52 ± 8.90%, respectively. Freeze-dried LNPs retained their basic physical properties and the three LNPs reducing KRAS^G12D^ mRNA levels by 58.47 ± 4.00, 51.83 ± 4.57, and 38.00 ± 4.89%, respectively.

**Discussion and conclusion:**

Natural lipids are promising components for LNPs construction, offering new avenues for siRNA delivery in gene therapy.

## Introduction

As a new therapeutic modality, RNA therapy has emerged as a critical technology in the global battle against COVID-19. Unlike small-molecule and antibody drugs targeting only 0.05% of the human genome, RNA drugs can selectively interact with proteins, transcripts, and genes, expanding the range of drug targets. RNA drugs offer the advantages of rapid design, low development costs, and negligible genotoxic effects, as they do not integrate into the genome (Kim 2022; Zhu et al. 2022). However, despite their promising development prospects, RNA therapies face challenges in delivery. RNA’s large molecular weight and negative charge contribute to electrostatic repulsion with negatively charged cell membranes, impeding its direct transmembrane transport to therapeutic sites. Furthermore, RNA’s inherent instability and susceptibility to degradation by RNA endonucleases in blood and tissues complicate its delivery to target sites, thereby limiting its therapeutic efficacy (Watts et al. 2008; Mousazadeh et al. 2021).

Advances in nanotechnology and related disciplines have addressed some of these delivery limitations. These drugs can be transported into cells *via* viral and non-viral vectors. Although viral vectors efficiently deliver RNA drugs, they pose safety risks due to their high immunogenicity and toxicity. In contrast, non-viral vectors, exemplified by lipid nanoparticles (LNPs), offer low immunogenicity, ease of synthesis, and improved safety (Miller 2003; Friedrich and Aigner 2022). However, inefficient lysosomal escape from RNA-loaded LNPs remains a significant bottleneck in their development (Hu et al. 2020; Herrera et al. 2021). Therefore, enhancing the lysosomal escape efficiency of LNPs is crucial to optimizing the current siRNA drug delivery.

Ionizable lipids, which have a pKa between 6 and 7, are essential components of contemporary LNP vector formulations. These lipids play a pivotal role in the dynamic transformation of intracellular lipid circulation and charge, effectively delivering siRNA (Han et al. 2021). However, synthesizing ionizable lipids is complex and expensive (Lou et al. 2020). To address these limitations, we explored the use of natural lipid components in developing LNPs to partially replace ionizable lipids, supplemented with synergistic cationic lipids. Natural lipids exhibit unique advantages, including cost-effectiveness, biocompatibility, and improved siRNA delivery efficiency. Natural lipids, such as *coix* seed and *Brucea javanica* seed lipids have already been successfully incorporated as primary components in commercial injections of traditional Chinese medicine (Fang et al. 2020; Zhang et al. 2021; Qin et al. 2023; Zhao et al. 2023). These natural lipids are biocompatible, exhibit low immunogenicity, and are cost-effective and abundantly available (Kim et al. 2016; Huang et al. 2019).

This study aimed to employ natural lipids as a component of LNPs to develop novel LNPs as siRNA delivery carriers. We examined the impact of the natural lipid ratio and the emulsifier ratio on the physical properties of LNPs (Z-average size, zeta potential, and polydispersity index). The cytotoxicity of LNPs was assessed by the MTS assay. The uptake efficiency, lysosomal escape, and co-localization with lipid droplets of FAM-siRNA-loaded LNPs were investigated. The transfection efficiency of LNPs was evaluated using PCR and Western blot (WB) assays. We also explored the freeze-drying conditions for LNPs and assessed mRNA and protein expression levels after freeze-drying and reconstitution.

## Materials and methods

### Materials

*Coix* seed lipid [*Coix lacryma-jobi* L. var. *mayuen* (Roman.) Stapf (Poaceae), CSL] (191117) was provided by Zhejiang Kanglaite Pharmaceutical Co., Ltd. Soybean oil [*Glycine max* (L.) Merr. (Fabaceae), SO] (S817900) was purchased from Shanghai McLean Biochemical Technology Co., Ltd. *Brucea javanica* seed lipid [*B. javanica* (L.) Merr. (Simaroubaceae), BJL] (FY36203) was purchased from Nantong Feiyu Biotechnology Co., Ltd. 1,2-dioleoyl-3-trimethylammonium-propane (DOTAP) (CG1086) and 1,2-dioleoyl-sn-3-phosphoethanolamine (DOPE) (FOE221024) were purchased from Aiweituo (Shanghai) Pharmaceutical Technology Co., Ltd. KRAS siRNA (A01001), KRAS^G12D^ siRNA (A01044) and FAM-siRNA (A07001) were purchased from Shanghai Jima Pharmaceutical Technology Co., Ltd. 4′,6-diamidino-2-phenylindole (DAPI) (S19119) was purchased from Shanghai Yuanye Biotechnology Co., Ltd. Hoechst33342 (C1022) and Lyso-Tracker Red (C1046) were purchased from Shanghai Biyuntian Biotechnology Co., Ltd. RPMI 1640 (C11875500BT), DMEM (C11995500BT), and penicillin-streptomycin (15140-122) were purchased from Gibco, USA. Fetal bovine serum (FBS) (086-150) was purchased from Vicente Biotechnology (Nanjing) Co., Ltd.

### Lipid nanoparticle formulations

DOTAP, DOPE, and one type of natural lipid (CSL, BJL, or SO) were mixed and dissolved in a 70 °C water bath to form the oil phase. Simultaneously, glycerol and water were combined and heated in a 70 °C water bath to prepare the aqueous phase. Once both phases reached the same temperature, the aqueous phase was gradually added to the oil phase under magnetic stirring. The prepared lipid nanoparticles (CSL-LNPs, BJL-LNPs, and SO-LNPs) were homogenized using a high-pressure homogenizer (FA25, FLUKO, China) at 20,000 rpm in 30-s on/off cycles for a total of 5 min. The nanoparticles were then subjected to ultrasonic treatment using a probe-type ultrasonic instrument (JY92-IIN, SCIENTZ, China) with a 2 mm diameter (30% amplitude, 2 s on and 2 s off intervals, for 8 min). CSL-LNPs, BJL-LNPs, and SO-LNPs were stored at 4 °C.

### Physicochemical characterization

The LNPs formulated with different components were diluted with deionized water at a 1:300 ratio. Z-average size, zeta potential, and PDI were measured using a Zetasizer Nano ZS instrument (Malvern Panalytical, UK) to determine the most effective formulation. The optimal LNP formulation was further diluted 100 times with deionized water and placed on a 300-mesh copper grid. After natural evaporation, the sample was negatively stained with 2% phosphotungstic acid. Transmission electron microscopy (TEM) was used to observe and capture images of the LNPs. TEM analysis was performed using a Hitachi H-7650 instrument (Hitachi, Japan).

### Agarose gel electrophoresis retardation experiment

To prepare the gel, 0.4 g of low-melting-point agarose was dissolved in 40 mL of 1 × TAE buffer by heating for 2 min until fully dissolved. After the solution was cooled slightly, 4 μL of GelRed nucleic acid dye was added and thoroughly mixed. The solution was then poured into an appropriate gel casting tray. Different N/P ratios were used to prepare corresponding LNP concentrations for co-incubation with siRNA. Once the gel solidified, the samples were loaded, and electrophoresis was conducted at 120 V for 15 min. The siRNA bands were visualized using a gel imaging system.

### Cell culture

The human pancreatic cancer cell line AsPC-1(CVCL_0152, CRL-1682) was purchased from the American Type Culture Collection (ATCC). The human embryonic kidney cell line HEK293T (CVCL_0063, CL-0005) was purchased from the Procell Life Science & Technology Co. Ltd. (Wuhan, China). The human normal pancreatic ductal epithelial cell line HPDE6-C7 (CVCL_0P38, H1-3201) was purchased from the Oricell Biotechnology Co. Ltd. (Guangzhou, China). AsPC-1 cells were cultured in RPMI-1640 medium supplemented with 10% FBS and 1% penicillin/streptomycin, then incubated at 37 °C in a 5% CO_2_ atmosphere. HPDE6-C7 and HEK293T cells were cultured in DMEM supplemented with 10% FBS and 1% penicillin/streptomycin under the same incubation conditions.

### Cell viability measurement

The cell viability of the three novel LNPs was assessed using an MTS assay. AsPC-1, HPDE6-C7, and HEK293T cells in the logarithmic growth phase were seeded in 96-well plates at an appropriate density. After incubation overnight at 37 °C in a 5% CO_2_ atmosphere, cells were treated with LNPs diluted in the medium at various concentrations and incubated for 48 h. The medium containing the drugs was discarded, and the cells were rinsed thoroughly with PBS (three times). Then, 100 μL of MTS reagent was added to each well, and the cells were incubated for an additional 2–3 h. After shaking for 20 s, the optical density (OD) was measured at 490 nm using a microplate reader. Cell viability was calculated using the formula: Cell viability % = (OD of drug-treated cells − OD of blank)/(OD of control cells − OD of blank) × 100%.

### Cell uptake

AsPC-1 cells in the logarithmic growth phase were seeded on cell slides at a density of 1 × 10^4^ cells per well and incubated overnight at 37 °C in a 5% CO_2_ atmosphere. Four hours before treatment, the medium was replaced with Opti-MEM medium for pretreatment. Lipo/FAM-siRNA complexes were prepared according to the manufacturer’s instructions. Similarly, CSL-LNPs, BJL-LNPs, and SO-LNPs were diluted in Opti-MEM medium and combined with FAM-siRNA at the same concentration of solution for subsequent use. The cells were rinsed twice with PBS and incubated with medium containing free FAM-siRNA, Lipo/FAM-siRNA, CSL-LNPs/FAM-siRNA, BJL-LNPs/FAM-siRNA, or SO-LNPs/FAM-siRNA (200 nM siRNA for each group) for 6 and 12 h. After incubation, the medium was discarded, and the cells were rinsed thoroughly with PBS (three times). The cells were then fixed with 4% paraformaldehyde (PFA) for 10 min, rinsed with PBS, and stained with 0.2 μg/mL DAPI at room temperature for 10 min. Following three additional PBS washes, the slides were sealed with an anti-fluorescence quenching solution. Cell uptake of FAM-siRNA was observed using a laser confocal microscope (Zeiss LSM880, Germany). The excitation and emission wavelengths (λ) for DAPI were 358 nm and 461 nm, respectively, while those for FAM were 494 nm and 519 nm, respectively.

### Lysosome escape of siRNA

AsPC-1 cells in the logarithmic growth phase were seeded in confocal dishes (1 × 10^4^ cells/dish) and incubated overnight at 37 °C in a 5% CO_2_ atmosphere. Four hours before treatment, the medium was replaced with Opti-MEM medium for pretreatment. The cells were washed twice with PBS phosphate buffer. Media containing Lipo/FAM-siRNA, CSL-LNPs/FAM-siRNA, BJL-LNPs/FAM-siRNA, and SO-LNPs/FAM-siRNA (200 nM siRNA for each group) were added, and cells were incubated for 6 and 12 h. Subsequently, the medium was removed, and the cells were washed three times with PBS. Cells were stained with Lyso-Tracker Red, preheated at 37 °C, for 30 min. The dye solution was removed. After washing three times with precooled PBS phosphate buffer, cells were stained with Hoechst 33342 nuclear dye (2 μg/mL) at room temperature for 10 min. Following three additional PBS washes, cells were examined under a laser confocal microscope. The excitation and emission wavelengths for Hoechst 33342 were 346 and 460 nm, respectively, while for Lyso-Tracker Red, they were 577 and 590 nm, respectively.

### Intracellular co-localization of siRNA and lipid droplets

AsPC-1 cells were seeded in a confocal dish (1 × 10^4^ cells/dish) and incubated overnight at 37 °C with 5% CO_2_. Four hours before treatment, the medium was replaced with Opti-MEM for pretreatment. Cells were washed twice with PBS, and 100 μL of Bodipy™ 558/568 c12 dye working solution was added. The dish was gently shaken to ensure complete coverage of the cells, and the cells were incubated for 10 min. After removing the dye solution, the cells were washed three times with PBS. Cells were then washed twice with PBS phosphate buffer, and media containing Lipo/FAM-siRNA, CSL-LNPs/FAM-siRNA, BJL-LNPs/FAM-siRNA and SO-LNPs/FAM-siRNA (200 nM siRNA for each group) was added and incubated for 12 h. Subsequently, the medium was discarded, and the cells were washed three times with PBS. Cells were fixed with 4% paraformaldehyde for 10 min, washed three times with PBS, and stained with 0.2 μg/mL DAPI solution at room temperature for 10 min. After three further washes with PBS, the cells were observed under a laser confocal microscope. The excitation and emission wavelengths for Bodipy™ 558/568 c12 were 558 and 568 nm, respectively.

### Quantitative real-time polymerase chain reaction (qRT-PCR)

Cells were seeded in 6-well plates at a density of 3 × 10^5^ cells/mL and incubated overnight at 37 °C. Four hours before transfection, the medium was replaced with serum-free medium. Prepared LNP-siRNA complex and Lipofectamine-siRNA complex were added to the wells. After 6 h of transfection, the medium was replaced with the complete medium. Total RNA was isolated from cells 48 h after treatment, and complementary DNA was synthesized using the Evo M-MLV reverse transcription premix kit (Cat # AG11728, Accurate Biology, China). Real-time RT-PCR was conducted using the SYBR Green Pro Taq HS premix qPCR kit (Cat # AG11718, Accurate Biology, China), which includes ROX, on the Applied Biosystems™ 7500 system. The gene expression levels of KRAS^G12D^ (forward 5′-ACTTGTGGTAGTTGGAGCAGA-3′, reverse 5′-TTGGATCATTCGTCCACAA-3′) and KRAS (forward 5′-CCCAGGTGCGGGAGAGA-3′, reverse 5′-CCCTCCCCAGTCC TCATGTA-3′) were compared to GAPDH (forward 5′-AGGTCGGTGTGAACGGATTTG-3′, reverse 5′-TGTAGACCATGTAGTTGAGGTCA-3′). Relative gene expression differences were calculated using the 2^−ΔΔCt^ method, where the amplification data were normalized to GAPDH expression(ΔCt = Ct(KRAS) − Ct(GAPDH)). Data from control (untreated) samples (ΔΔCt = ΔCt (siKRAS-treated samples) − ΔCt (untreated samples)) were set as a baseline, with 100% expression, and the results from other groups were standardized against this control for comparison.

### Protein extraction, gel electrophoresis, and Western blot assay

Cells were seeded in 6-well plates at 3 × 10^5^ cells/mL density and incubated overnight at 37 °C in a 5% CO_2_ atmosphere. Four hours before transfection, the medium was replaced with serum-free medium. The prepared LNPs-siRNA complex and Lipofectamine-siRNA were added to the wells. After 6 h of transfection, the medium was replaced with a complete medium. Total protein was isolated from cells 72 h after treatment, and its concentration was determined using the BCA protein assay kit (Biosharp, BL521A). The protein loading amount was 50 μg/well. The proteins were separated by sodium dodecyl sulfate-polyacrylamide gel electrophoresis (SDS-PAGE) and transferred onto a polyvinylidene fluoride (PVDF) membrane. The membrane was blocked with 5% skim milk for 2 h and incubated overnight at 4 °C with primary antibodies. The primary antibodies used were: Anti-KRAS^G12D^ (Human, 1:1000, AB_2877649, ab221163, Abcam), Anti-KRAS (Human, 1:1000, AB_3071781, HA601059, Huabio), Anti-GAPDH (Human, 1:2000, AM8539b, Abcepta). The membrane was incubated with secondary antibodies. The secondary antibodies used were: Goat Anti-Rabbit IgG (1:2000, RS23920, Immunoway), Goat Anti-Mouse IgG (1:2000, RS23710, Immunoway) at room temperature for 2h. Detection was performed using the LI-COR Odyssey Clx near-infrared imaging system.

### Freeze drying of natural LNPs

Freeze-drying protective agents (mannitol, glucose, sucrose) were added to the oil phase at mass ratios ranging from 1:2 to 1:10. LNPs without any protective agents served as a blank control group. The LNP-protective agent mixtures were vortexed until the agents dissolved completely. The mixture was pre-frozen at −20 °C for 12 h, then vacuum-freeze dried for 48 h. After freeze drying, the effectiveness was assessed by examining the changes in the Z-average size and PDI upon reconstitution, which helped determine the optimal ratio of oil phase to protective agent.

The freeze-dried LNPs were redissolved in an equal volume of deionized water and characterized by the Z-average size, the zeta potential, the PDI, and the TEM. The binding efficacy of LNPs and siRNA after reconstitution was confirmed by an agarose gel electrophoresis retardation assay. mRNA expression levels were validated by PCR, and protein expression levels were assessed by WB.

### Statistical analysis

All data are presented as mean ± standard deviation (mean ± *SD*). Statistical analyses were conducted using the GraphPad Prism 5 software. The significance level (alpha) was set at 0.05. Statistical differences are as follows: *p < 0.05, **p < 0.01, ***p < 0.001, ^****^p < 0.0001, and ns for insignificant.

## Results

### Effects of natural lipids and auxiliary lipids on the physicochemical characterization of LNPs

**Figure 1.**
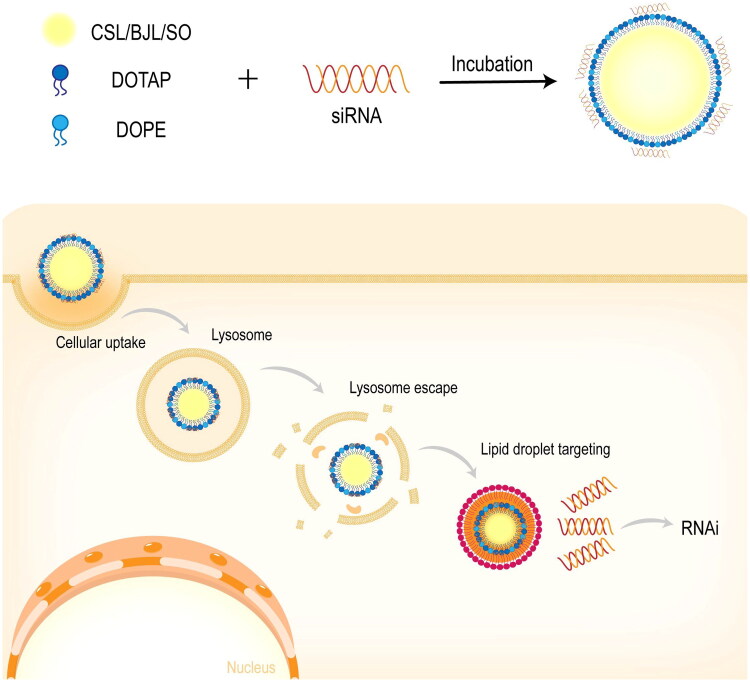
Construction of natural lipid LNPs and schematic diagram for siRNA delivery.

Three siRNA delivery formulations were developed based on different natural lipids. These formulations included two types of traditional Chinese medicine lipids (CSL and BJL) and a natural lipid (SO), along with other components, such as DOTAP, DOPE, glycerol, and deionized water (Figure 1).

We conducted a formulation screening to obtain LNPs with optimal physical characteristics, such as the Z-average size and PDI. The results indicated that the content of natural lipids, DOTAP, and DOPE in the formulation significantly affected the physical properties of LNPs. The optimal formulation consisted of 3.52% natural lipids, 0.35% DOTAP, 0.12% DOPE, 2.11% glycerol, and 93.9% deionized water. The Z-average diameters of CSL-LNPs, BJL-LNPs, and SO-LNPs in this optimal formulation were found to be 170.6 ± 0.3, 164.8 ± 2.3, and 187.2 ± 2.4 nm, respectively, as determined by a Malvern laser particle size analyzer. The surface potential ranged between 44 and 50 mV, and the PDI was <0.2, indicating high stability (Tables 1–3).

**Table 1.** CSL-LNPs formulation screening.

Formulation	CSL	DOTAP	DOPE	PDI	Size (nm)	Zeta (mV)
1	1.79%	0.35%	0.12%	0.168	250.0 ± 2.6	47.2
2	3.52%	0.35%	0.12%	0.112	170.6 ± 0.3	45.8
3	6.80%	0.35%	0.12%	0.259	412.7 ± 5.5	48.7
4	15.26%	0.35%	0.12%	0.427	639.1 ± 9.5	48.4
5	3.52%	0.18%	0.06%	0.197	327.3 ± 3.4	45.8
6	3.52%	1.05%	0.12%	0.184	299.8 ± 3.1	53.4

**Table 2.** SO-LNPs formulation screening.

Formulation	SO	DOTAP	DOPE	PDI	Size (nm)	Zeta (mV)
1	1.79%	0.35%	0.12%	0.166	268.0 ± 1.8	47.4
2	3.52%	0.35%	0.12%	0.099	187.2 ± 2.4	44.7
3	6.80%	0.35%	0.12%	0.215	373.7 ± 2.7	44.1
4	15.26%	0.35%	0.12%	0.305	430.3 ± 9.7	43.6
5	3.52%	0.18%	0.06%	0.217	343.0 ± 3.5	42.7
6	3.52%	1.05%	0.12%	0.313	465.7 ± 6.8	42.9

**Table 3.** BJL-LNPs formulation screening.

Formulation	BJL	DOTAP	DOPE	PDI	Size (nm)	Zeta (mV)
1	1.79%	0.35%	0.12%	0.188	272.5 ± 2.5	49.3
2	3.52%	0.35%	0.12%	0.174	164.8 ± 2.3	49.8
3	6.80%	0.35%	0.12%	0.348	383.9 ± 6.0	50.5
4	15.26%	0.35%	0.12%	0.408	699.5 ± 17.2	56.3
5	3.52%	0.18%	0.06%	0.250	317.7 ± 5.8	47.1
6	3.52%	1.05%	0.12%	0.187	224.4 ± 2.4	44.3

**Figure 2.**
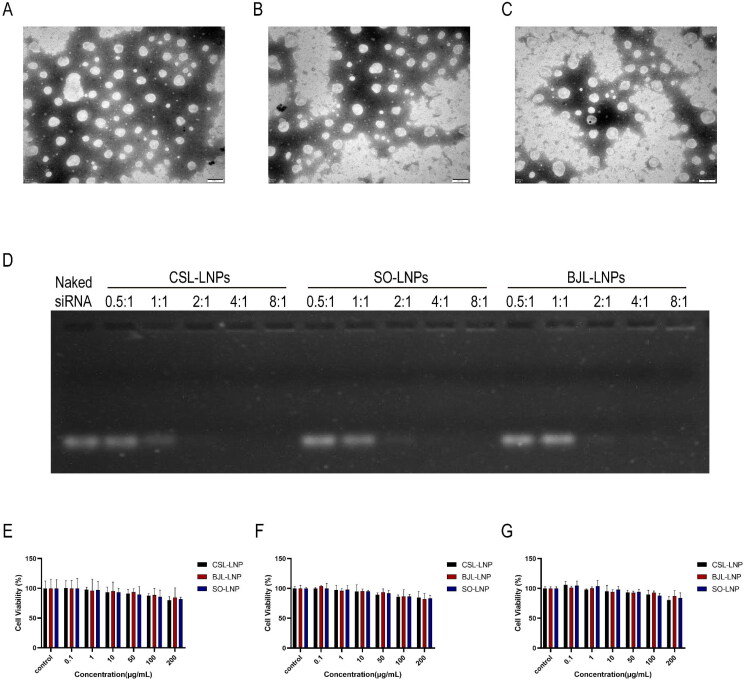
Construction and characterization of three natural LNPs. (A) TEM images of CSL-LNPs. (B) TEM images of SO-LNPs. (C) TEM images of BJL-LNPs. (D) siRNA gel retardation experiments for the three natural LNPs, categorized by N/P ratios: 0.5:1, 1:1, 2:1, 4:1, and 8:1. (E) Effects of the three natural LNPs on cell viability in AsPC-1 cells. (F) Impact on cell viability in HPDE6-C7 cells. (G) Influence on cell viability in HEK293Tcells.

TEM revealed that the three LNPs were spherical and well dispersed (Figures 2(A–C)). According to the optimal formulation, siRNA was added to the LNPs. The corresponding concentration of LNPs was prepared and combined with siRNA in different N/P ratios. The ability of the LNPs to bind to siRNA was further assessed by agarose gel retardation experiments. The results did not show free siRNA in the three groups when the N/P ratio was 2:1 (Figure 2(D)). This demonstrates that the three LNPs, based on different natural lipids, can completely adsorb siRNA in this N/P ratio, with no observed difference in adsorption capacity.

Subsequently, we used AsPC-1, HPDE6-C7, and HEK293T cells for our research. First, we explored the cytotoxicity of newly prepared LNPs in these three cell types. The results indicated that within the 0–200 μg/mL concentration range, the cell survival rate was ≥80% when the LNP concentration reached 200 μg/mL (Figures 2(E–G)). When the LNP concentration was ≤50 μg/mL, the cell survival rates were >90%. These findings demonstrate that the toxicity of the three new LNPs was consistent across all three cell types, with no significant change in cell viability within the concentration range tested. Therefore, the new LNPs exhibit high safety and are suitable for use as transfection agents.

The chemical constituents of CSL, BJL, and SO were analyzed by gas chromatography-mass spectrometry (GC/MS). The chemical structure of each component was determined according to the standard spectral library and the relevant literature (Figures S1 and S2) and the composition and relative content of fatty acids in CSL, BJL and SO were calculated by area normalization method. The results showed that 13 fatty acid components were detected in CSL, BJL, and SO, of which 8 saturated fatty acids and 5 unsaturated fatty acids were detected in CSL and SO. BJL detected 6 saturated fatty acids and 6 unsaturated fatty acids. The relative content of each component group showed some differences (Table S1).

### Intracellular uptake and lysosomal escape of natural lipid nanoparticles

**Figure 3.**
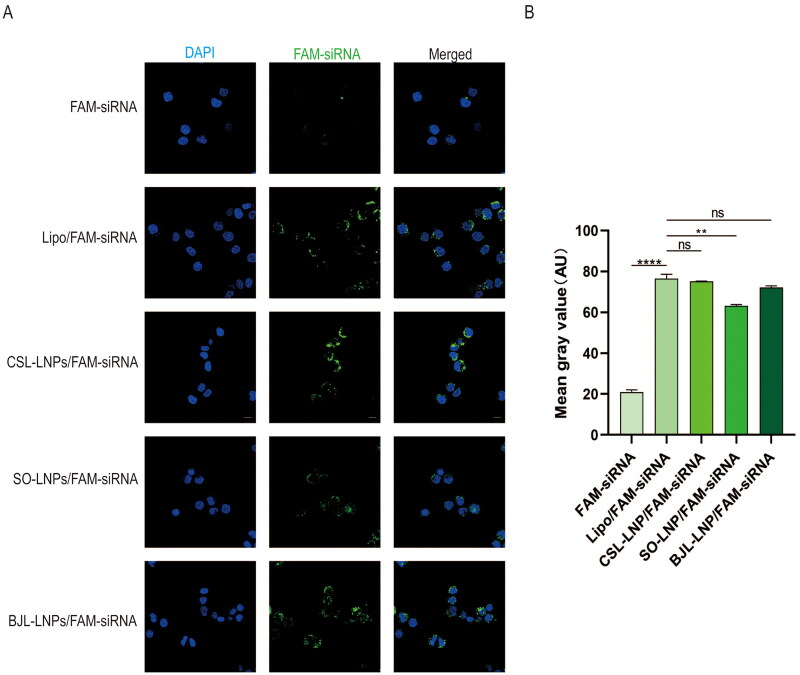
Uptake efficiency of FAM-siRNA in AsPC-1 cells. (A) Representative confocal images showing cellular uptake of free FAM-siRNA, Lipo/FAM-siRNA, CSL-LNPs/FAM-siRNA, SO-LNPs/FAM-siRNA, and BJL-LNPs/FAM-siRNA after 6 h of co-incubation with AsPC-1 cells. (B) Quantitative map of mean fluorescence intensity for the groups shown in (A). *Note:* ns means no significant difference, **P < 0.01, ****P < 0.0001.

To verify the cellular uptake efficiency of the prepared natural lipid nanoparticles, we used CLSM to observe changes in intracellular fluorescence intensity after co-incubation of AsPC-1 cells with free FAM-siRNA, Lipo/FAM-siRNA, CSL-LNPs/FAM-siRNA, BJL-LNPs/FAM-siRNA and SO-LNPs/FAM-siRNA. The results indicated that the fluorescence intensity in the CSL-LNPs/FAM-siRNA, BJL-LNPs/FAM-siRNA, and SO-LNPs/FAM-siRNA groups was significantly higher than in the free FAM-siRNA group, with a substantial number of FAM fluorescence signals observed in cells (Figures 3(A,B)). Additionally, the fluorescence signal values in the CSL-LNPs/FAM-siRNA, BJL-LNPs/FAM-siRNA, and Lipo/FAM-siRNA groups were similar, indicating that the LNPs and Lipo had comparable transfection efficiency and could effectively deliver siRNA into cells.

**Figure 4.**
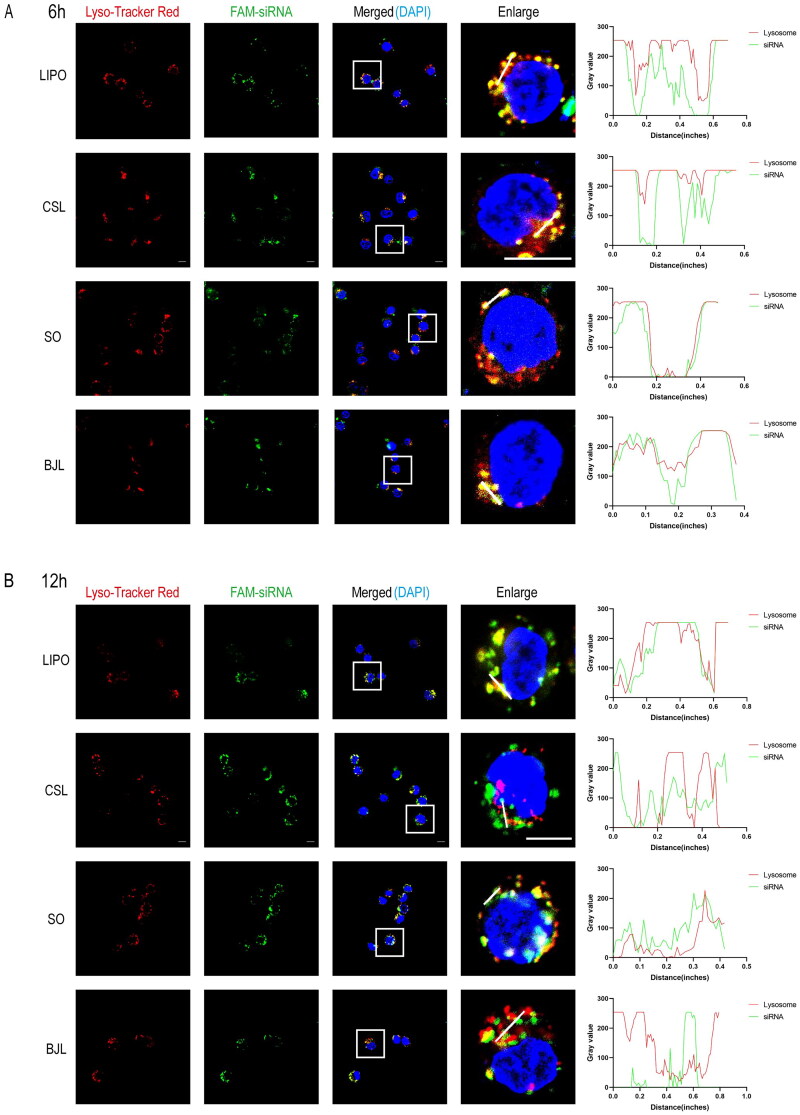
Lysosomal escape of Lipo/FAM-siRNA, CSL-LNPs/FAM-siRNA, SO-LNPs/FAM-siRNA, and BJL-LNPs/FAM-siRNA in AsPC-1 cells. (A) Representative confocal intracellular co-localization images and representative fluorescence co-localization analysis curves of AsPC-1 cells co-incubated with Lipo/FAM-siRNA, CSL-LNPs/FAM-siRNA, SO-LNPs/FAM-siRNA, and BJL-LNPs/FAM-siRNA for 6 h. Lysosome (Lyso-Tracker red stained lysosomes showed red fluorescence), FAM-siRNA (green fluorescence), DAPI (blue fluorescence of the nucleus). Scale, 20 μm. (B) Representative intracellular confocal co-localization images and representative fluorescence co-localization analysis curves of AsPC-1 cells co-incubated with Lipo/FAM-siRNA, CSL-LNPs/FAM-siRNA, SO-LNPs/FAM-siRNA, and BJL-LNPs/FAM-siRNA for 12 h. Lysosome (Lyso-Tracker red stained lysosomes showed red fluorescence), FAM-siRNA (green fluorescence), DAPI (blue fluorescence of the nucleus). Scale, 20 μm.

After entering the cell, siRNA must escape from the lysosome to enter the cytoplasm and silence the gene post-transcription. To explore lysosomal escape after LNP-mediated siRNA delivery, we conducted a lysosome escape experiment. Lipo, CSL-LNPs, BJL-LNPs, and SO-LNPs carrying FAM-siRNA were incubated with AsPC-1 cells for 6 h, and the results of co-localization were consistent. The results showed that most of the green fluorescence emitted by FAM-siRNA overlapped with the red fluorescence emitted by the lysosomes, resulting in a yellow signal (Figure 4(A)). This indicated that the carriers entered the lysosome through endocytosis and that it indicated that FAM-siRNA had not completely escaped the lysosome at 6 h. When the incubation time was extended to 12 h (Figure 4(B)), most of the green fluorescence in the CSL-LNPs and BJL-LNPs groups was separated from the red fluorescence, indicating that the siRNA mainly escaped the lysosome and was successfully delivered. In the Lipo and SO-LNPs groups, only part of the green fluorescence was separated from the red fluorescence.

### Natural lipid nanoparticles loaded with siRNA co-localized with lipid droplets

**Figure 5.**
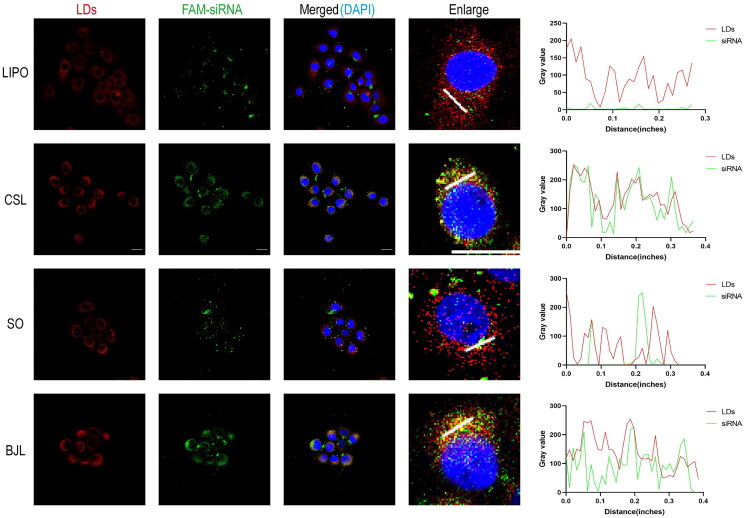
The co-localization of Lipo/FAM-siRNA, CSL-LNPs/FAM-siRNA, SO-LNPs/FAM-siRNA, and BJL-LNPs/FAM-siRNA with lipid droplets in AsPC-1 cells. Representative confocal intracellular co-localization images and representative fluorescence co-localization analysis curves of AsPC-1 cells co-incubated with Lipo/FAM-siRNA, CSL-LNPs/FAM-siRNA, SO-LNPs/FAM-siRNA, and BJL-LNPs/FAM-siRNA for 12 h. LDs (Bodipy™ 558/568 c12 stained lipid droplets showed red fluorescence), FAM-siRNA (green fluorescence), DAPI (blue nucleus fluorescence). Scale, 20 μm.

As mentioned above, the lipid droplets were labeled with Bodipy™ 558/568 C12, and the FAM-siRNA was adsorbed by LNPs for co-localization experiments. The results showed strong overlapping yellow signals of FAM green fluorescence and Bodipy™ 558/568 C12 red fluorescence in the CSL-LNPs and BJL-LNPs groups, indicating that FAM-siRNA adsorbed by these groups had a more pronounced co-localization with lipid droplets (Figure 5). In contrast, there was no apparent co-localization in the Lipo and SO-LNPs groups. These results suggest that traditional Chinese medicine lipids (CSL and BJL) have a natural lipid droplet-targeting capability compared to natural lipids (SO), which can mediate enhanced lysosomal escape of siRNA.

### Transfection rate of natural lipid nanoparticles

Over the past few decades, KRAS has been considered a ‘non-pharmaceutical’ target, with relatively few drugs directly targeting the KRAS gene. Therefore, we designed a study using siRNA interference to down-regulate KRAS expression.

**Figure 6.**
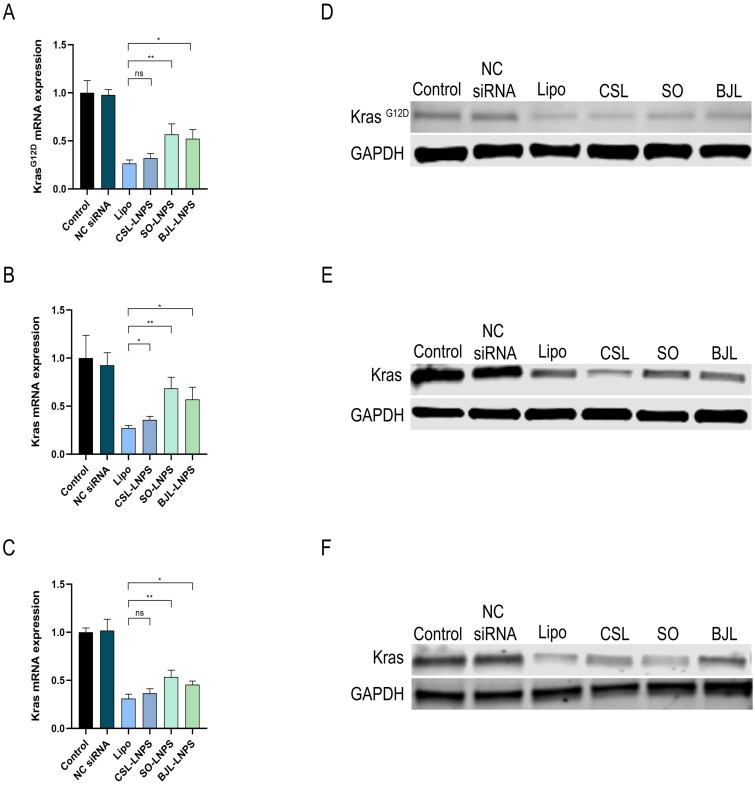
The transfection efficiency of LNPs based on different natural lipids in three cell lines measured by qRT-PCR and Western blotting. (A) Quantitative PCR was used to measure KRAS^G12D^ expression in AsPC-1 cells. (B) Quantitative PCR was used to measure the expression of KRAS in HPDE6-C7 cells. (C) Quantitative PCR was used to measure the expression of KRAS in HEK293T cells. (D) Western blot was used to detect the level of KRAS^G12D^ in AsPC-1 cells. (E) Western blot was used to compare the level of KRAS in HPDE6-C7 cells. (F) Western blot was used to compare the level of KRAS in HEK293T cells. *Note:* ns means no significant difference, *P < 0.05, **P < 0.01.

The transfection results in AsPC-1 cells (Figures 6(A,D)) showed that all-natural LNPs successfully mediated siRNA delivery to knock down the KRAS^G12D^ gene and protein levels. CSL-LNPs, BJL-LNPs, and SO-LNPs reduced KRAS^G12D^ mRNA levels in AsPC-1 cells by 67.87 ± 3.89, 47.18 ± 7.65, and 42.52 ± 8.90%, respectively. There was no significant difference in the knockdown efficiency of CSL-LNPs compared to the positive control vector Lipo, which reduced KRAS^G12D^ mRNA levels by 73.43 ± 2.87%. The knockdown efficiency of BJL-LNPs and SO-LNPs was lower than that of Lipo but still achieved 64.25 and 57.91%, respectively.

The transfection results in HPDE6-C7 and HEK293T cells (Figures 6(B,C,E,F)) also showed that all natural LNPs could successfully mediate siRNA delivery to knock down KRAS gene and protein levels. In HPDE6-C7 cells, CSL-LNPs, BJL-LNPs, and SO-LNPs reduced KRAS mRNA levels by 64.31 ± 3.59, 43.10 ± 9.82, and 31.51 ± 8.61%, respectively. KRAS mRNA levels in HEK293T cells decreased by 63.46 ± 4.69, 54.58 ± 3.71, and 46.52 ± 7.10%, respectively. For HPDE6-C7 cells, the knockdown efficiency of CSL-LNPs, BJL-LNPs, and SO-LNPs was 88.52, 59.33, and 43.37%, respectively, compared to the positive control vector Lipo, which reduced KRAS mRNA by 72.65 ± 2.45%. For HEK293T cells, the transfection results were similar to those of AsPC-1 cells. The knockdown efficiency of CSL-LNPs was not significantly different from that of Lipo. Although the knockdown efficiency of BJL-LNPs and SO-LNPs was lower than that of Lipo, they still reached 79.02 and 67.35%, respectively.

### Freeze-dried protective agent affects the physicochemical characterization of freeze-dried LNP products

**Figure 7.**
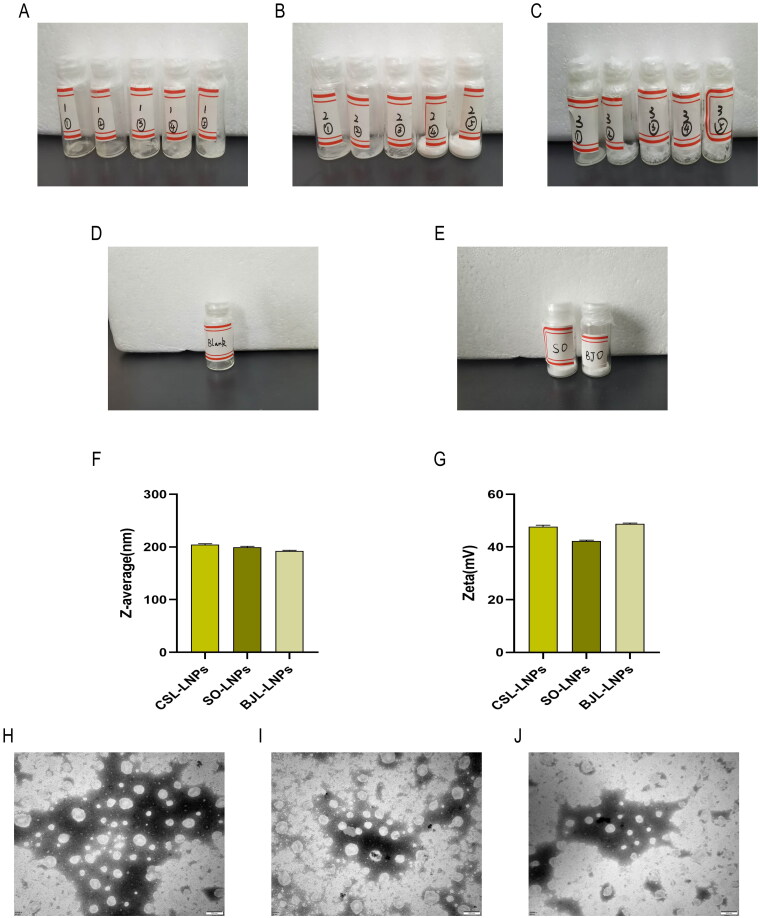
Characterization of LNPs freeze-dried products. (A) Mannitol as a freeze-dried protective agent. The mass ratio of oil phase to mannitol from left to right is: 1:2, 1:4, 1:6, 1:8, and 1:10. (B) Glucose as a freeze-dried protective agent. The mass ratio of the oil phase to glucose from left to right is: 1:2, 1:4, 1:6, 1:8, and 1:10. (C) Sucrose as a freeze-dried protective agent. The mass ratio of oil phase to sucrose from left to right is: 1:2, 1:4, 1:6, 1:8, and 1:10. (D) Freeze-dried appearance of CSL-LNPs without a freeze-dried protective agent. (E) Appearance of so-LNPs and BJL-LNPs after freeze-drying. (F) Particle size of three LNPs after freeze-drying. (G) Zeta potential of three LNPs after freeze-drying. (H) CSL-LNPs after freeze-drying (TEM). (I) SO-LNPs after freeze-drying (TEM). (J) BJL-LNPs after freeze-drying (TEM).

We screened three freeze-drying protective agents—mannitol, glucose, and sucrose—and examined the mass ratio of the oil phase to the freeze-drying protective agent. Sixteen samples of 0.4 mL CSL-LNPs were taken in 5 mL vials. The freeze-drying protective agent was added according to a specific mass ratio of the oil phase and the freeze-drying protective agent, and the mixture was vortexed to dissolve completely. A sample without a freeze-drying protective agent was used as the blank control group. The samples were frozen at −20 °C for 12 h, followed by vacuum freeze-drying for 48 h. The appearance of the freeze-dried LNPs (Figures 7(A–D)) was observed, and the Z-average size and PDI after freeze-drying were measured (Table 4) to evaluate the characteristics of the freeze-dried LNPs.

**Table 4.** Effects of the ratio of freeze-dried protective agent, oil phase, and freeze-dried protective agent on the particle size and PDI of CSL-LNPs.

Formulation	Freeze-drying protectant	The proportion of oil phase and freeze-dried protective agent	Z-average size (nm)	PDI
1	Blank		953.4 ± 48.6	0.898
2	Mannitol	1:2	430.3 ± 7.7	0.440
3		1:4	394.8 ± 20.7	0.386
4		1:6	376.7 ± 5.0	0.346
5		1:8	265.2 ± 4.1	0.227
6		1:10	254.8 ± 5.0	0.179
7	Glucose	1:2	315.9 ± 4.2	0.310
8		1:4	267.2 ± 2.8	0.198
9		1:6	265.2 ± 0.5	0.190
10		1:8	204.4 ± 1.9	0.180
11		1:10	199.4 ± 1.7	0.130
12	Sucrose	1:2	301.5 ± 6.3	0.242
13		1:4	276.7 ± 10.1	0.280
14		1:6	271.0 ± 6.8	0.236
15		1:8	218.8 ± 2.8	0.143
16		1:10	199.0 ± 1.4	0.120

The blank control group (Figure 7(D)), which did not contain a freeze-drying protective agent, showed a yellow oily appearance, with the Z-average size and PDI significantly increasing to 953.4 ± 48.6 nm and 0.898, respectively (Table 4). When glucose was used as the freeze-drying protective agent, with an oil phase to protective agent ratio of 1:8, the appearance of the freeze-dried CSL-LNPs was ideal white, smooth, and full (Figure 7(B)), and the Z-average size and PDI did not increase significantly, measuring 204.4 ± 1.9 nm and 0.180, respectively (Table 4). Using mannitol as the freeze-drying protective agent resulted in a collapsed appearance of CSL-LNPs, with significant increases in Z-average size and PDI (Figure 7(A)). Sucrose as a freeze-drying protective agent did not result in a significant rise in Z-average size and PDI compared to pre-freeze-drying, but the appearance of the freeze-dried products did not meet expectations (Figure 7(C)). Therefore, an oil phase to freeze-drying protective agent ratio of 1:8 was selected, with glucose as the optimal freeze-drying protective agent.

After determining the optimal ratio of the oil phase to the freeze-drying protective agent and identifying the most suitable protective agent, we applied these freeze-drying conditions to BJL-LNPs and SO-LNPs. These conditions resulted in an ideal snow white, smooth, and full appearance for BJL-LNPs and SO-LNPs (Figure 7(E)). There was no significant change in Z-average size and potential (Figures 7(F,G)). The Z-average size and potential of BJL-LNPs were 192.5 ± 1.1 nm and 48.8 ± 0.3 mV, respectively. For SO-LNPs, the Z-average size and potential were 199.37 ± 1.7 nm and 42.2 ± 0.4 mV, respectively.

Freeze drying can potentially alter the morphology of the nanocarriers, so we observed the three LNPs after freeze-drying using electron microscopy (Figures 7(H–J)). The three LNPs preserved their spherical morphology after freeze-drying, consistent with their morphology before freeze-drying. Electron microscopy confirmed that the freeze-drying conditions stably preserved the LNPs without compromising their morphological characteristics.

### siRNA adsorption capacity and in vitro transfection efficiency of LNPs after lyophilization

**Figure 8.**
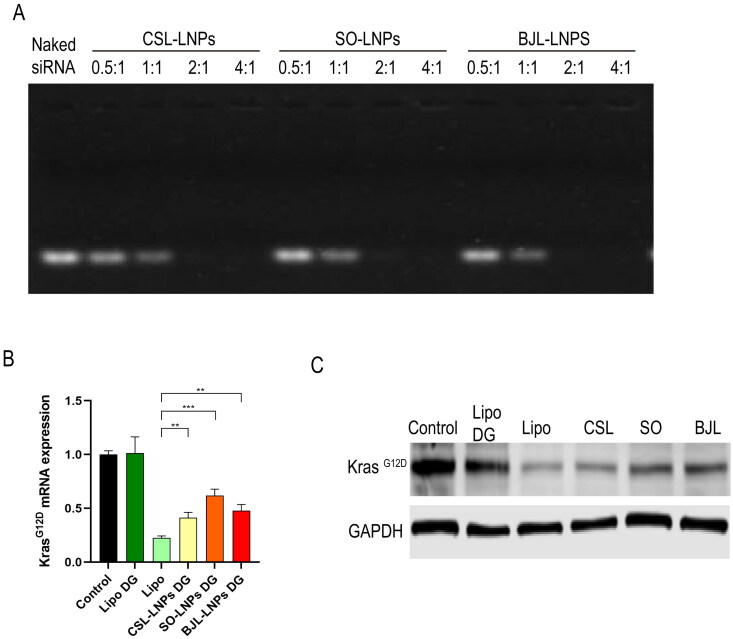
The *in vitro* transfection efficiency of the three LNPs after lyophilization was evaluated. (A) siRNA gel retardation experiments for the three natural LNPs after lyophilization, categorized by N/P ratios: 0.5:1, 1:1, 2:1, 4:1. (B) The transfection efficiency of freeze-dried LNP (AsPC-1 cells) was detected by qRT-PCR. (C) Western blot was used to detect the protein expression level of each group. *Note:* **P < 0.01, ***P < 0.001.

We performed agarose gel retardation, PCR, and WB experiments on freeze-dried LNPs to investigate their ability to adsorb siRNA and their *in vitro* transfection efficiency. After re-dissolution, the three LNPs retained a strong ability to adsorb siRNA, completely adsorbing siRNA at an N/P ratio of 2:1 (Figure 8(A)). This demonstrated that their siRNA adsorption capacity remained unchanged after freeze-drying.

Finally, we evaluated the *in vitro* transfection efficiency of the three LNPs after lyophilization. The experiment included a normal control group, a positive control Lipo group, an experimental group (freeze-dried CSL-LNPs, SO-LNPs, and BJL-LNPs), and a freeze-dried Lipo group. Freeze-dried CSL-LNPs and BJL-LNPs demonstrated significant transfection efficiency, reducing KRAS^G12D^ mRNA levels by 58.47 ± 4.00 and 51.83 ± 4.57%, respectively. The transfection efficiency of SO-LNPs was 38.00 ± 4.89%. In contrast, the commercially available Lipo transfection reagent showed almost no transfection ability after freeze-drying (Figure 8(B)). The WB results were consistent with those of PCR (Figure 8(C)), further confirming that the novel LNPs retained their ability to transfect siRNA effectively after lyophilization and reconstitution.

## Discussion

The natural LNPs prepared in this study include CSL-LNPs, BJL-LNPs, and SO-LNPs, all of which exhibit favorable physical and chemical characteristics. The Z-average sizes of CSL-LNPs, BJL-LNPs, and SO-LNPs were 170.6 ± 0.3, 164.8 ± 2.3, and 187.2 ± 2.4 nm, respectively. The surface potential ranged between 44 and 50 mV, and the PDI was <0.2, indicating high stability. Additionally, the N/P ratio of the three natural lipid nanoparticles was 2:1, demonstrating a high RNA adsorption capacity. These physicochemical characteristics are comparable to those of commonly used cationic LNPs for small nucleic acid delivery (Gregersen et al. 2024; Nabar et al. 2024; Zhao et al. 2024).

*In vitro* studies revealed that the natural LNPs exhibited high siRNA transfection efficiency both before and after lyophilization, with CSL-LNPs showing higher transfection efficiency than BJL-LNPs and SO-LNPs. Furthermore, the transfection efficiency of CSL-LNPs was comparable to that of the commercial transfection reagent Lipo. Our results confirm that natural lipids from traditional Chinese medicine, such as CSL and BJL, can be used to construct LNPs for siRNA delivery. These natural LNPs effectively overcome challenges associated complex preparation, high cost, and low biocompatibility found in synthetic lipids, such as ionizable cationic lipids and cationic lipids. Additionally, they address the issue of high stability required for the storage of conventional LNPs.

Various types of lipids, such as ionizable lipids, cationic lipids, solid lipid nanoparticles (SLNs), and nanostructured liposomes (NLCs), are widely used in the development of LNPs for small nucleic acid delivery (Tenchov et al. 2021; Viegas et al. 2023; Wu et al. 2024). These LNPs have the advantages of high nucleic acid encapsulation efficiency, effective cell transfection, strong tissue penetration, low cytotoxicity, and immunogenicity, making them excellent candidates for nucleic acid delivery systems (Gómez-Aguado et al. 2020; Aldosari et al. 2021; Chatzikleanthous et al. 2021). However, some lipids or surfactants used in LNPs may have potential toxicity and immunogenicity, and the current synthetic lipid chemistry is complex, expensive, and less sustainable (Azarnezhad et al. 2020).

However, natural lipids have good quality control, biocompatibility, and human safety. CSL, extracted from *coix* seed, has various pharmacological activities (Manosroi et al. 2016). The main component of CSL, *C. lacryma-jobi* L. var. *mayuen* (Roman.) Stapf (Poaceae) seed oil, *is a* 2,3-butanediol ester of unsaturated fatty acids and has been shown to have anti-tumor, antioxidant, and immune-stimulating effects (Ukita and Tanimura 1961; Xi et al. 2016; Hou et al. 2018). BJL is a complex mixture of fatty acids and fatty acid derivatives extracted from the fruit of *B. javanica* (L.) Merr. (Ma et al. 2013). It is one of the main active ingredients, with anti-inflammatory, anti-malarial, and anti-tumor activities (Su et al. 2002; Pan et al. 2009; Chen et al. 2015). Fatty oil-SO, extracted from the leguminous plant Glycine soja Bentham seeds, is widely used as an oil phase for the preparation of lipid emulsions due to its rich resources and high compatibility. It is used in marketed drugs, such as aprepitant emulsion and propofol medium- and long-chain fat emulsion (Xu et al. 2023).

These natural lipids have been used as injection-grade pharmaceutical raw materials. For example, Kanglaite injection, a microemulsion product derived from CSL, is widely used in treating various tumors (Lu et al. 2008). Natural lipid-based nanocarriers have demonstrated enhanced drug delivery efficacy. A microemulsion prepared with CSL as an oil phase could improve the solubility of the poorly soluble drug triterpene and improve its efficacy against lung cancers (Qu et al. 2014). Chen et al. assembled *Tripterygium wilfordii* and *coix* seed oil into a two-component microemulsion system to treat cervical cancer (Chen et al. 2018).

We used the advantages of natural lipids to prepare LNPs for siRNA delivery. The results of cell-level transfection experiments showed that the transfection efficiency of CSL-LNPs was not significantly different from that of commercially available carrier Lipo, with mRNA expression levels reduced by 63.46–67.87%. Although the transfection efficiency of BJL-LNPs and SO-LNPs was lower than that of Lipo, they still reached 79.02 and 67.35% of Lipo’s efficiency, respectively. A key factor influencing the transfection performance of these nanocarriers is their lysosomal escape capability.

LNPs enter cells primarily through macropinocytosis and clathrin-mediated endocytosis and then fuse with lysosomes. However, due to the low lysosomal escape efficiency of LNPs, siRNA drugs often fail to reach the cytoplasm efficiently to exert their therapeutic effects. Studies have shown that LNP-delivered siRNA is still mainly enriched in the liver, with only 1–2% of internalized LNP-loaded siRNA being released into the cytoplasm, and this release occurs within a limited time after internalization (Sahay et al. 2013; Wittrup et al. 2015). Therefore, improving lysosomal escape efficiency is crucial for effective siRNA delivery by LNPs.

The cell uptake efficiency of natural LNPs was different. The cell uptake efficiency of CSL-LNPs and BJL-LNPs was higher than that of SO-LNPs and comparable to the commercial transfection reagent Lipo. The lysosomal escape experiment showed that the green fluorescence of the CSL-LNPs and BJL-LNPs groups mostly escaped from the red fluorescence at 12 h, indicating that siRNA successfully escaped from the lysosome. In contrast, only part of the green fluorescence was separated from the red fluorescence in the Lipo and SO-LNPs groups. The results showed that the lysosomal escape efficiency of SO-LNPs was lower than that of CSL-LNPs and BJL-LNPs.

Since *coix* seed oil has a natural lipid droplet-targeting property, we hypothesized that it enhances the lysosomal escape of siRNA. We designed a co-localization experiment of siRNA and lipid droplets to test this hypothesis. The results showed strong co-localization of FAM-siRNA green fluorescence with lipid droplet red fluorescence in the CSL-LNPs and BJL-LNPs groups, indicated by the strong yellow fluorescence signal. In contrast, the Lipo and SO-LNPs groups showed weak yellow fluorescence signals with no apparent co-localization. This indicates that CSL-LNPs and BJL-LNPs can target lipid droplets in cells, whereas Lipo and SO-LNPs cannot.

Several studies have shown that delivery carriers containing *coix* seed oil as a lipid component can achieve lysosomal escape in cells and play an anti-tumor role in coordination with other drugs (Guo et al. 2019, 2021). These findings demonstrate that lipid droplet targeting facilitates lysosomal escape of natural lipid LNPs.

The development of LNPs products on the market is currently hampered by complex construction components, unique preparation processes, and strict preservation conditions (Wang et al. 2020; Li et al. 2022; Kon et al. 2023; Wang et al. 2023). Due to their limited thermal stability, LNPs require ultra-low temperature storage conditions. The core of the mRNA-loaded LNP contains a significant amount of water, which may be a crucial factor in reducing the biological activity of mRNA LNPs due to hydrolysis and colloidal instability when stored under environmental conditions (Lamoot et al. 2023). Therefore, freeze-drying is a logical and attractive method for improving the thermal stability of these LNPs.

To enhance the long-term storage stability of the prepared LNPs, we freeze-dried them. We chose glucose as the freeze-drying protective agent because it maintains the Z-average size and PDI within a small range and provides a better freeze-drying appearance. With glucose as the freeze-drying protective agent, the Z-average size of the freeze-dried CSL-LNPs, BJL-LNPs, and SO-LNPs increased by 10–15 nm but maintained a PDI of <0.2 and the same potential value.

*In vitro* transfection experiments showed that the freeze-dried LNP products still maintained a certain level of transfection efficiency. The levels of KRAS^G12D^ mRNA knocked down by CSL-LNPs and BJL-LNPs were 58.47 and 51.83%, respectively, while the level of KRAS^G12D^ mRNA knocked down by SO-LNPs was 38.00%. However, the commercially available lipotransfection reagent showed almost no transfection ability after lyophilization, possibly due to changes in the integrity and composition of the liposome after the freeze-drying process.

The three natural lipids, CSL, BJL, and SO, have shown potential for constructing LNPs for small nucleic acid delivery. Compared with BJL and SO, CSL has better siRNA delivery efficiency. However, we need to expand the research scope to identify more natural lipids that can be used to build small nucleic acid delivery vectors. However, this study did not include *in vivo* verification of natural lipid LNPs. Subsequent research should focus on the *in vivo* siRNA delivery efficiency, the characterization of the opsonization, and underlying delivery mechanism of the natural lipid LNPs.

## Conclusions

LNPs made from natural lipids like CSL, BJL and SO displayed sizes between 160 and 180 nm with a PDI under 0.2 and stable potential, suggesting superior physical properties. These LNPs, comparable in cell uptake and transfection efficiency to commercial Lipo, maintained effectiveness even after freeze-drying. Their enhanced safety, stability, and lysosomal escape efficiency facilitate siRNA delivery into the cytoplasm for gene therapy. Using natural lipids offers new avenues for developing diverse LNP formulations and methods for delivering small nucleic acid drugs, warranting further exploration in therapeutic applications.

## Supplementary Material

Supplementary material.docx

## Data Availability

The datasets used and/or analyzed are available from the corresponding author upon reasonable request.
