# Supplementary material for: Delivery of small interfering RNA through lyophilized natural lipid nanoparticles: effects of natural lipid selection
Source: Pharm Biol. 2025 May 2;63(1):343–56. doi: 10.1080/13880209.2025.2498169 (PMC12051533; doi:10.1080/13880209.2025.2498169)
Supplement: Supplementary material.docx [file IPHB_A_2498169_SM4341.docx]

**Materials and methods**

***Materials***

Potassium hydroxide (P112287), sodium chloride (A67512) and 14 % boron trifluoride methanol (B822337) were purchased by Hangzhou Bangyi Chemical Co., Ltd. Methanol (MS1922-801) was purchased by Shanghai Scientific Instrument Co., Ltd. Hexane (H810749) was purchased by Shanghai Tengzhun Biotechnology Co., Ltd.

***Methods***

The main fatty acids composition of Coix seed lipid, Brucea javanica seed lipid and Soybean oil were qualitatively characterized according to the following methods(Uzun et al. 2008; Deivajothi et al. 2019). The samples of Coix seed lipid, Brucea javanica seed lipid and Soybean oil were taken and accurately weighed 0.1g, and placed in 10 mL test tube with plug scale, respectively. Then 2 mL of 0.8 mol/L potassium hydroxide methanol solution were added in a water bath thermostatic oscillator at 80°C water bath for 20 min, cooled to room temperature. Add 2 mL of 14% boron trifluoride methanol, shake, bath in water at 80°C for 15 min, take out and cool to room temperature. Precisely add 2 mL of n-hexane and shake well. Add 2 mL saturated sodium chloride solution, stand and layer, take the supernatant.

Gas chromatographic conditions(Awogbemi et al. 2019) : chromatographic column was HP-5MS capillary column ( 30 m × 0.25 mm × 0.25 μm ). The heating program was maintained at 80°C for 0 min. The temperature was raised from 20°C/min to 145°C and maintained for 0 min. Then it was increased to 220°C at a rate of 5°C/min for 5 min. Finally, the temperature was raised to 280°C at a rate of 10°C/min for 5 min. Injector temperature : 250°C, carrier gas flow rate :1 mL/min, splitting ratio: 1 : 250.

Mass spectrometry conditions: ion source temperature(Ma et al. 2024) : 200°C, transmission line temperature: 250°C, solvent delay time: 2.5 min, mass scanning range: 40-600 amu, ion source: EI source 70 eV. Agilent 7890-5977A gas chromatography-single quadrupole mass spectrometer was produced by Agilent Technology Co., Ltd.

**References**

Awogbemi O, Onuh EI, Inambao FL. 2019. Comparative study of properties and fatty acid composition of some neat vegetable oils and waste cooking oils. International Journal of Low-Carbon Technologies. 14(3):417-425. doi: 10.1093/ijlct/ctz038.

Deivajothi P, Manieniyan V, Sivaprakasam S. 2019. Experimental investigation on DI diesel engine with fatty acid oil from by-product of vegetable oil refinery. Ain Shams Engineering Journal. 10(1):77-82. doi: 10.1016/j.asej.2018.04.005.

Ma Y-J, Li P, Zhu B-W, Du M, Xu X-B. 2024. Comprehensive determination of fatty acids in real samples without derivatization by DMU-SPME-GC methods. Food Research International. 195:114986. doi: 10.1016/j.foodres.2024.114986.

Uzun B, Arslan Ç, Furat Ş. 2008. Variation in Fatty Acid Compositions, Oil Content and Oil Yield in a Germplasm Collection of Sesame (Sesamum indicum L.). Journal of the American Oil Chemists' Society. 85(12):1135-1142. doi: 10.1007/s11746-008-1304-0.


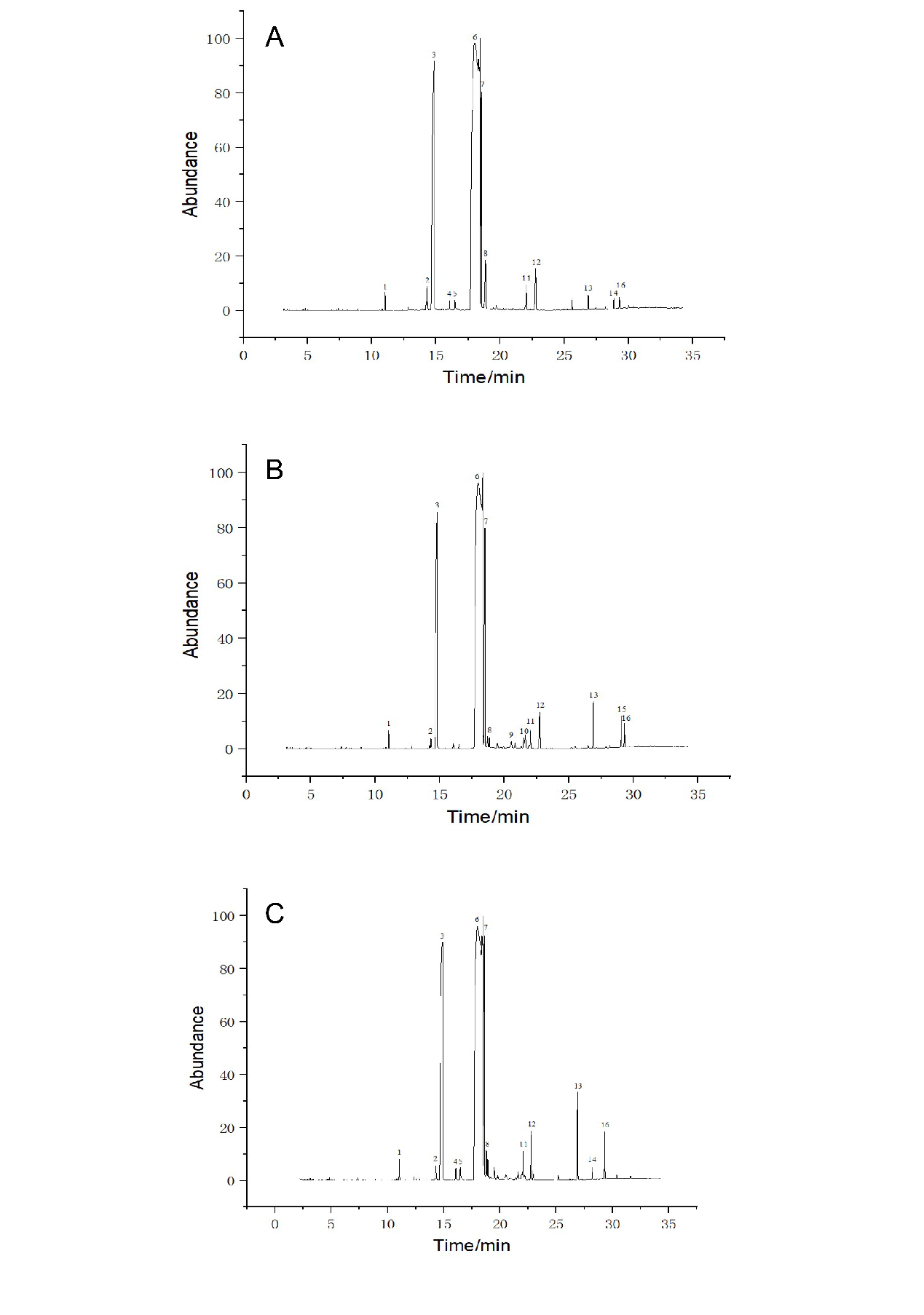
Figure S1. GC/MS analysis of fatty acid components in CSL, BJL and SO. (A) GC/MS analysis of CSL fatty acid composition. (B) GC/MS analysis of BJL fatty acid composition. (C) GC/MS analysis of SO fatty acid components.


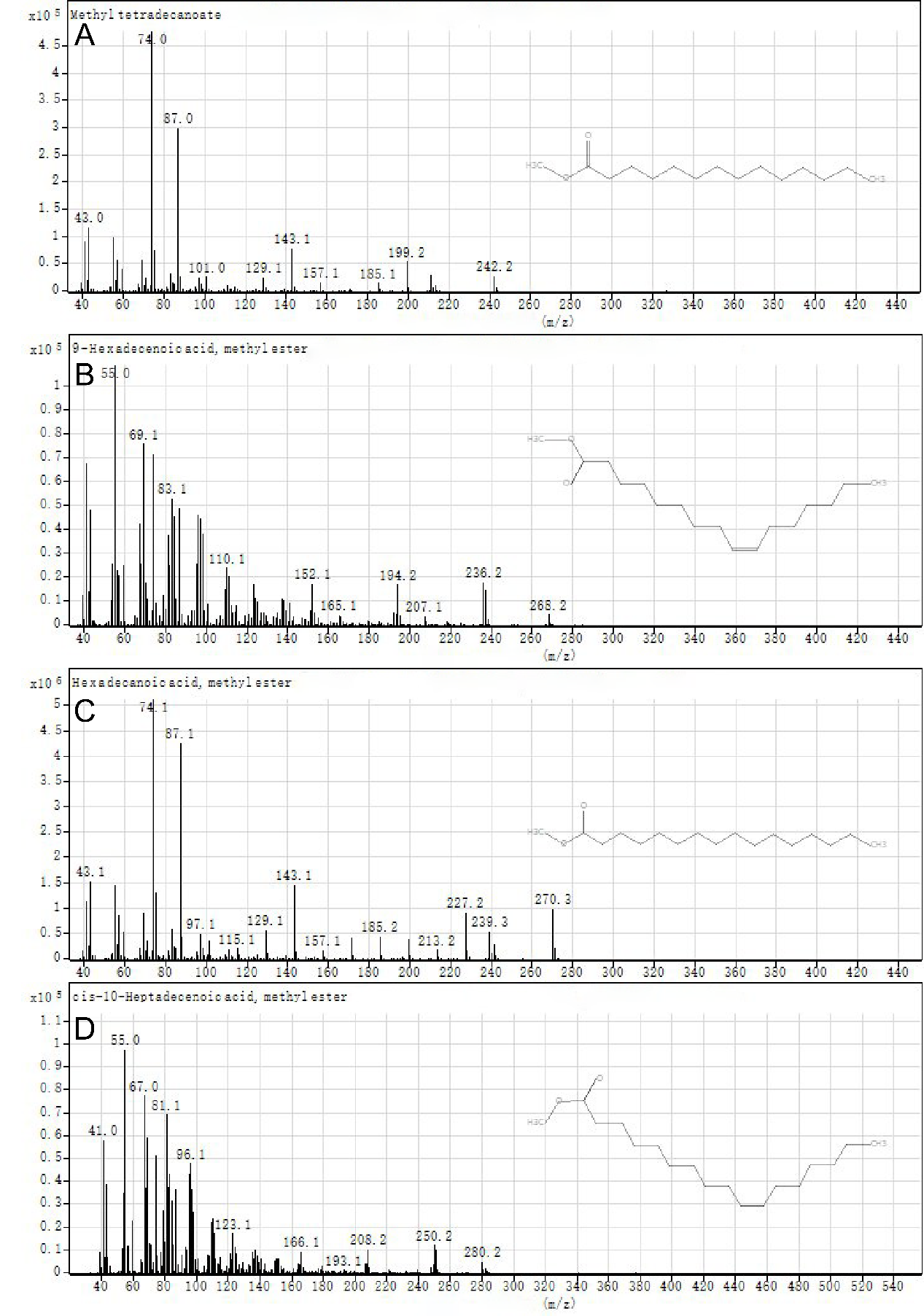


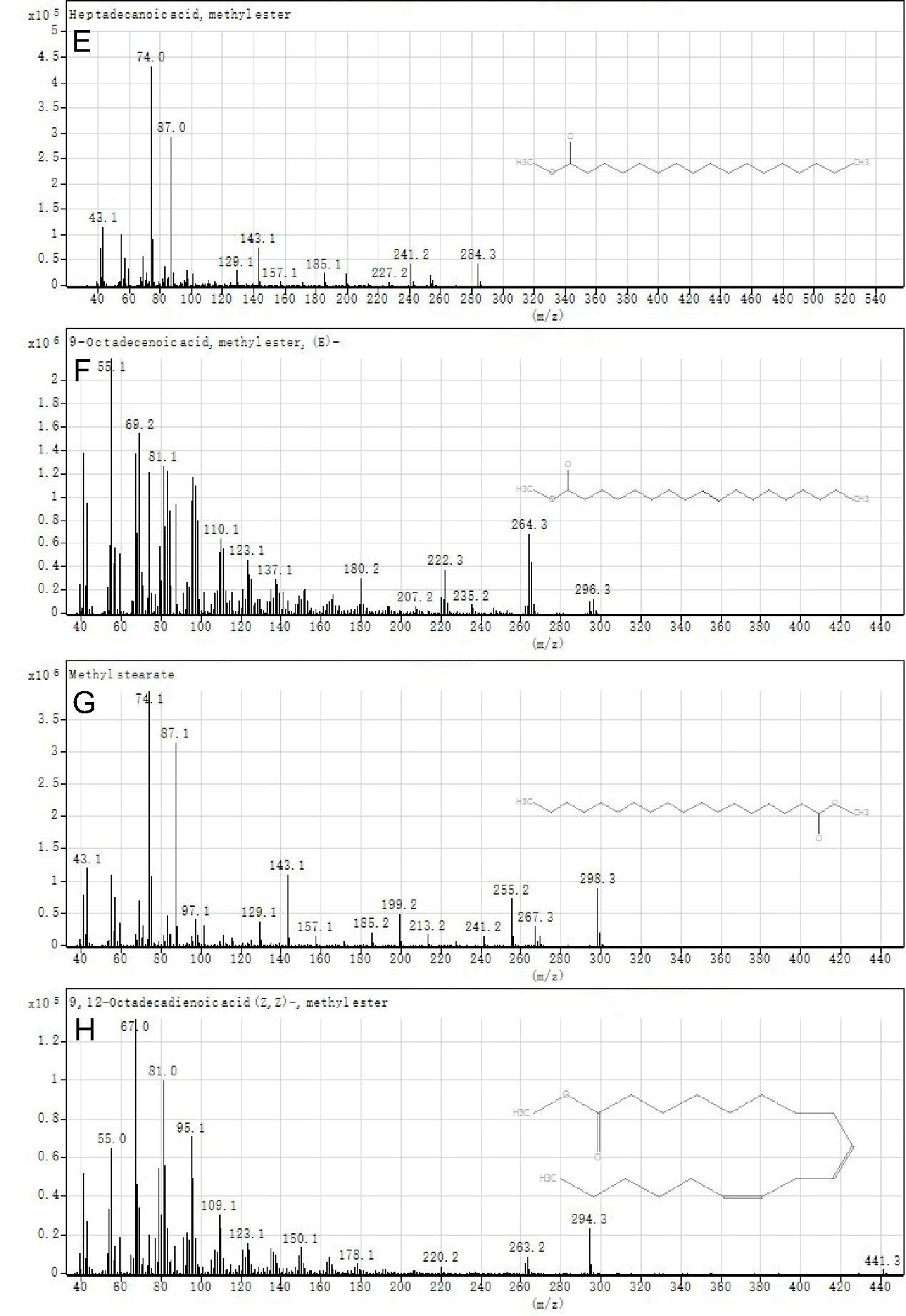


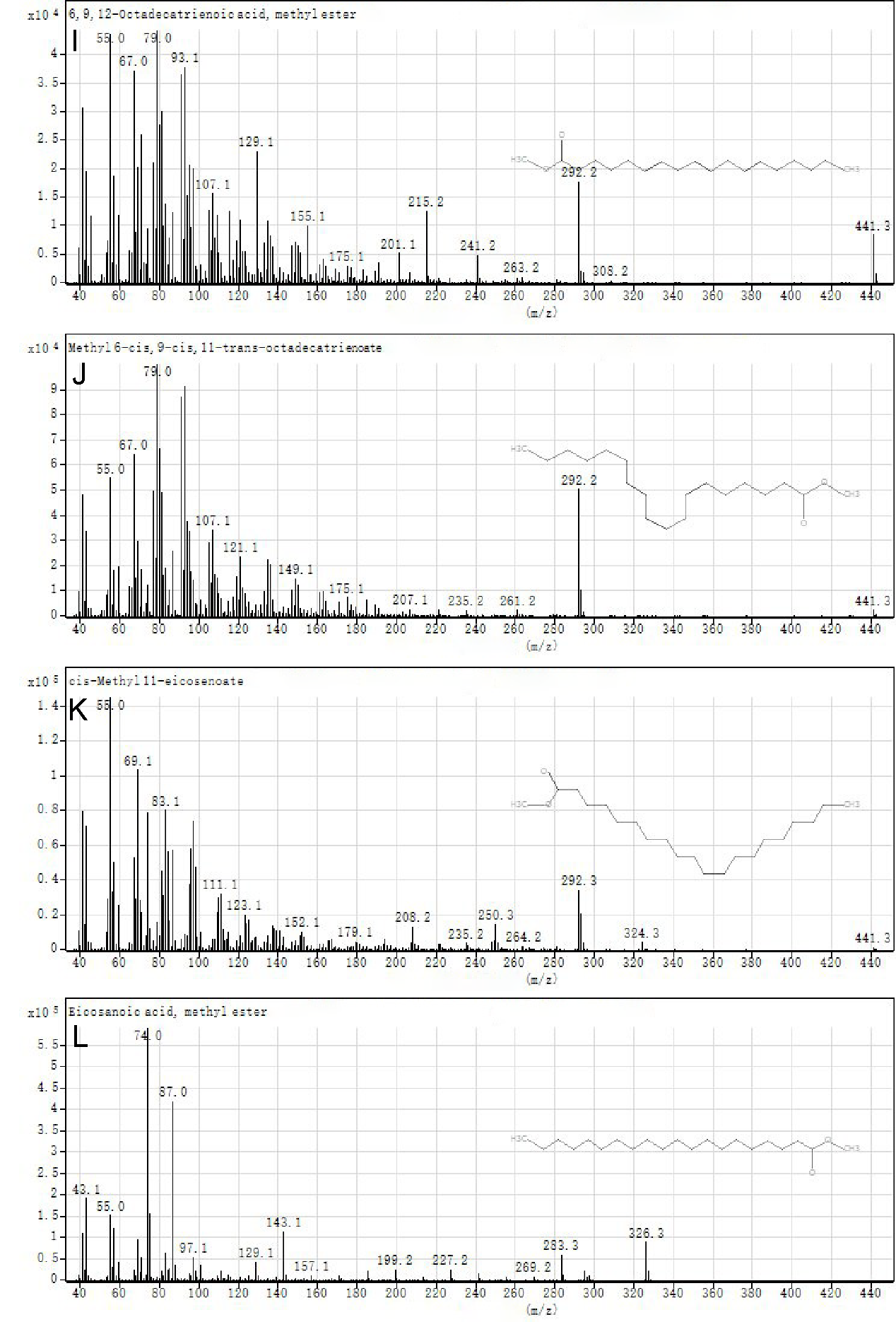

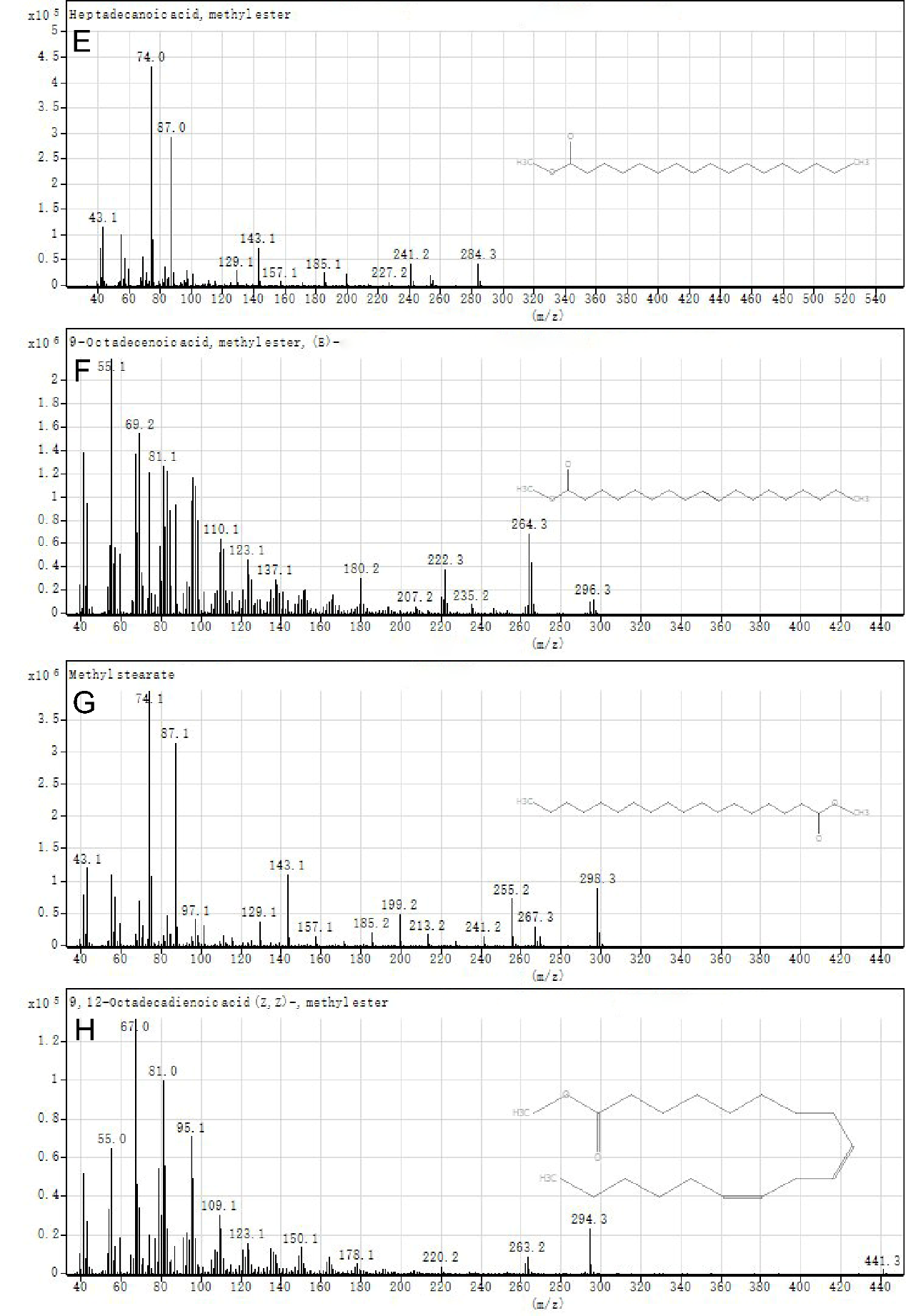


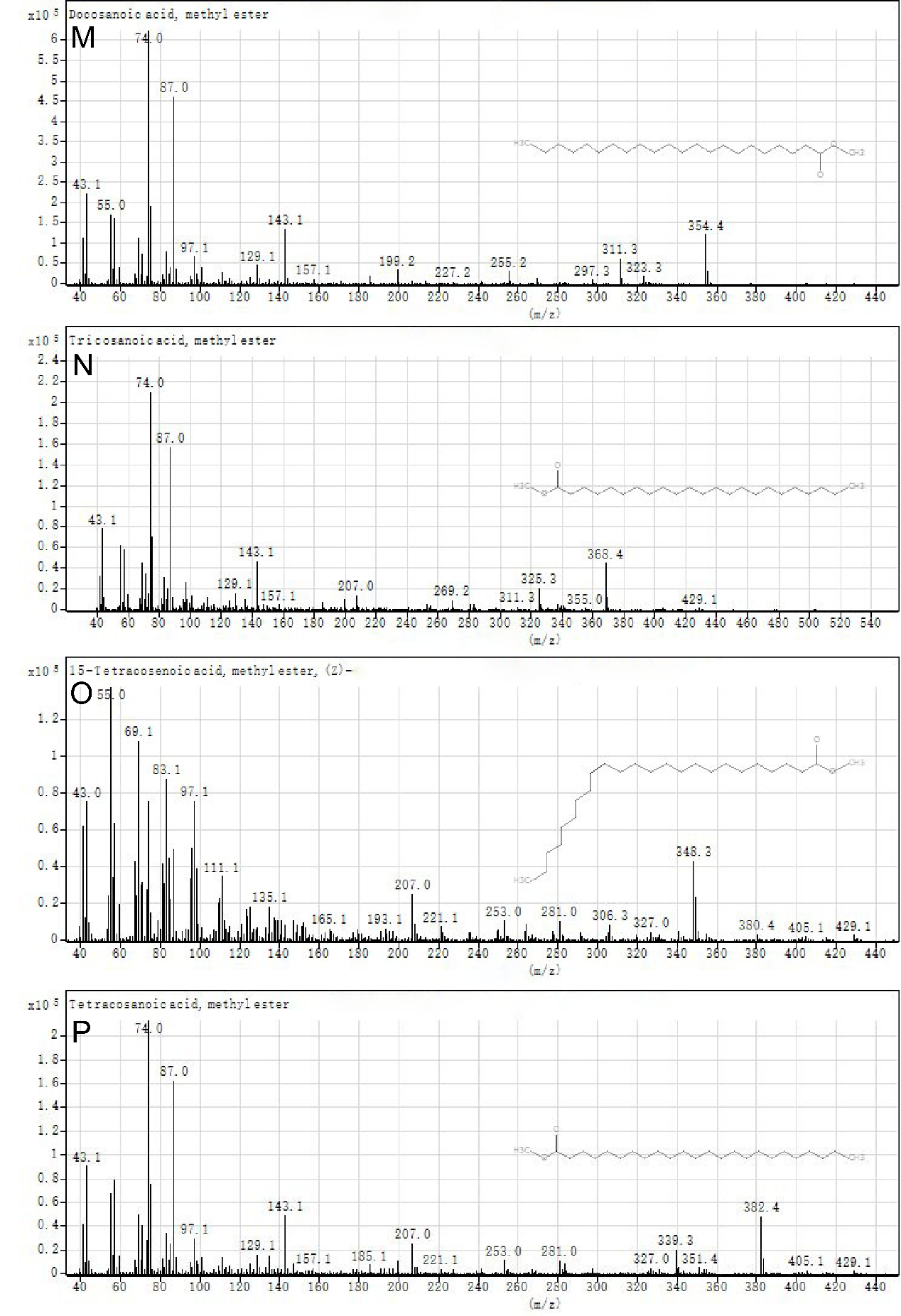
Figure S2. Mass spectra of 16 fatty acids. (A) Tetradecanoic acid (B) 9- Hexadecenoic acid (C) Hexadecanoic acid (D) Cis-10-Heptadecenoic acid (E) Heptadecanoic acid (F) 9-Octadecenoic acid (G) Stearic acid (H) 9,12-Octadecadienoic acid (I) 6,9,12-Octadecatrienoic acid (J) 6-cis,9-cis,11-trans-Octadecatrienoic acid (K) cis-11-Eicosenoic acid (L) Eicosanoic acid (M) Docosanoic acid (N) Tricosanoic acid (O) 15-Tetracosenoic acid (P) Tetracosanoic acid

**Table S1.** The relative content of the main compounds of the three oils

| Index | RT/min | | | Compound | Chemical Formula | Average Relative Content/% | | |
| --- | --- | --- | --- | --- | --- | --- | --- | --- |
|  | CSL | BJL | SO |  |  | CSL | BJL | SO |
| 1 | 11.258 | 11.051 | 11.051 | Tetradecanoic acid | C14H28O2 | 0.34 | 0.32 | 0.28 |
| 2 | 14.310 | 14.292 | 14.301 | 9-Hexadecenoic acid | C16H30O2 | 0.58 | 0.24 | 0.34 |
| 3 | 14.877 | 14.793 | 14.888 | Hexadecanoic acid | C16H32O2 | 19.37 | 13.67 | 17.92 |
| 4 | 16.063 | —— | 16.057 | cis-10-Heptadecenoic acid | C17H32O2 | 0.23 | —— | 0.26 |
| 5 | 16.477 | —— | 16.470 | Heptadecanoic acid | C17H34O2 | 0.28 | —— | 0.33 |
| 6 | 18.029 | 17.952 | 18.011 | 9-Octadecenoic acid | C18H34O2 | 69.62 | 74.09 | 67.10 |
| 7 | 18.543 | 18.495 | 18.613 | Stearic acid | C18H36O2 | 5.36 | 6.89 | 8.62 |
| 8 | 18.862 | 18.825 | 18.790 | 9,12-Octadecadienoic  acid | C18H32O2 | 1.51 | 0.22 | 0.65 |
| 9 | —— | 20.555 | —— | 6,9,12-Octadecatrienoic acid | C18H30O2 | —— | 0.37 | —— |
| 10 | —— | 21.612 | —— | 6-cis，9-cis，11-trans-Octadecatrienoic acid | C18H30O2 | —— | 0.44 | —— |
| 11 | 22.044 | 22.031 | 22.055 | cis-11-Eicosenoic acid | C20H38O2 | 0.81 | 0.63 | 0.79 |
| 12 | 22.764 | 22.745 | 22.775 | Eicosanoic acid | C20H40O2 | 1.33 | 1.28 | 1.37 |
| 13 | 26.872 | 26.878 | 26.895 | Docosanoic acid | C22H44O2 | 0.26 | 0.94 | 1.57 |
| 14 | 28.844 | —— | 28.188 | Tricosanoic acid | C23H46O2 | 0.16 | —— | 0.18 |
| 15 | —— | 29.044 | —— | 15-Tetracosenoic acid | C24H46O2 | —— | 0.53 | —— |
| 16 | 29.293 | 29.292 | 29.298 | Tetracosanoic acid | C24H48O2 | 0.15 | 0.39 | 0.60 |

Note : - means not detected.
